# A lightweight and efficient model for grape bunch detection and biophysical anomaly assessment in complex environments based on YOLOv8s

**DOI:** 10.3389/fpls.2024.1395796

**Published:** 2024-08-06

**Authors:** Wenji Yang, Xiaoying Qiu

**Affiliations:** Software College, Jiangxi Agricultural University, Nanchang, China

**Keywords:** grape bunches detection, YOLOv8, lightweight, slim-neck, NSPPF

## Abstract

As one of the most important economic crops, grapes have attracted considerable attention due to their high yield, rich nutritional value, and various health benefits. Identifying grape bunches is crucial for maintaining the quality and quantity of grapes, as well as managing pests and diseases. In recent years, the combination of automated equipment with object detection technology has been instrumental in achieving this. However, existing lightweight object detection algorithms often sacrifice detection precision for processing speed, which may pose obstacles in practical applications. Therefore, this thesis proposes a lightweight detection method named YOLOv8s-grape, which incorporates several effective improvement points, including modified efficient channel attention (MECA), slim-neck, new spatial pyramid pooling fast (NSPPF), dynamic upsampler (DySample), and intersection over union with minimum point distance (MPDIoU). In the proposed method, MECA and NSPPF enhance the feature extraction capability of the backbone, enabling it to better capture crucial information. Slim-neck reduces redundant features, lowers computational complexity, and effectively reuses shallow features to obtain more detailed information, further improving detection precision. DySample achieves excellent performance while maintaining lower computational costs, thus demonstrating high practicality and rapid detection capability. MPDIoU enhances detection precision through faster convergence and more precise regression results. Experimental results show that compared to other methods, this approach performs better in the grapevine bunch detection dataset and grapevine bunch condition detection dataset, with mean average precision (mAP50–95) increasing by 2.4% and 2.6% compared to YOLOv8s, respectively. Meanwhile, the computational complexity and parameters of the method are also reduced, with a decrease of 2.3 Giga floating-point operations per second and 1.5 million parameters. Therefore, it can be concluded that the proposed method, which integrates these improvements, achieves lightweight and high-precision detection, demonstrating its effectiveness in identifying grape bunches and assessing biophysical anomalies.

## 1 Introduction

Grapes are not only delicious but also highly nutritious, boasting a high yield (Restani et al., 2021). With an annual production of approximately 7.5 million tons, grapes are cultivated worldwide, with 41% of production in Europe, 29% in Asia, and 21% in the USA (Colombo et al., 2019). Grapes are widely used in various industries, including winemaking, fresh consumption, and food processing. About 50% of grapes are used in wine production, one-third as fresh fruit, and the remainder is refined to produce grape jams, grape juice, grape seed oil, and various other grape-based products (Kandylis et al., 2021). The global wine industry, valued in billions of dollars, encompasses a wide range of economic activities (Arnó et al., 2009). Vineyards require careful management to balance grape quality and quantity, maximizing profitability in wine production (Bramley, 2010). To achieve accurate and timely yield estimation and enhance quality, it is essential to closely monitor grape bunches throughout the growing season and perform timely pruning and fruit thinning to prevent an excessive burden on the plant (Li et al., 2023). Disease management is also a significant concern in the grape and wine industry (Renouf and Lonvaud-Funel, 2007), and managers in vineyards need to assess biophysical anomaly assessment. In the past, grape management typically relied on manual methods, which were time-consuming and labor-intensive. Therefore, it is crucial to find solutions that enable farmers to produce with high quality, higher yields, and lower costs. Automated equipment holds promising prospects for this endeavor (Tang et al., 2020; Zhou et al., 2022). By combining automated machinery with state-of-the-art (SOTA) object detection technology, automatic identification of grape bunches and biophysical anomaly assessment are achieved, thereby fulfilling the economic goals pursued by farmers.

In recent years, deep learning (DL) has had a significant impact on the development of computer vision in artificial intelligence (LeCun et al., 2015). Convolutional neural networks (CNNs) in DL have been widely employed in the field of agriculture and have shown superiority over existing conventional image processing techniques (Kamilaris and Prenafeta-Boldú, 2018a; Santos et al., 2020; Kamilaris and Prenafeta-Boldú, 2018b). During the establishment of smart orchards, the use of object detection to identify targets and diagnose diseases contributes to intelligent orchard management, ultimately improving crop yield and quality. Object detection can be categorized into those based on classical machine learning (ML) and those based on DL (Zhao et al., 2022). However, the former demands manual feature engineering, necessitating personnel with high levels of expertise and experience, and is susceptible to the complexities of the environment (Chen et al., 2023). With the advancement of DL, the precision, speed, and robustness of the latter surpass the former (Liang et al., 2020). Generally, object detection based on DL can be categorized into two-stage and one-stage detection methods. The difference lies in the fact that the former involves proposing a set of candidate regions (region proposal) before regressing their positions and classifying the candidate regions. In contrast, the latter eliminates the region proposal stage, directly predicting bounding boxes and computing class probabilities for these boxes (Sirisha et al., 2023). Classic two-stage algorithms include R-CNN (Girshick et al., 2014), Fast R-CNN (Girshick, 2015), and Faster R-CNN (Ren et al., 2015). One-stage algorithms include the You Only Look Once (YOLO) series (Redmon et al., 2016; Redmon and Farhadi, 2017; Redmon and Farhadi, 2018; Bochkovskiy et al., 2020; Ultralytics, 2020; Li et al., 2022; Wang et al., 2022; Ultralytics, 2023), Single-Shot Multi-Box Detector (SSD) (Liu et al., 2016), CenterNet (Duan et al., 2019), and RetinaNet (Lin et al., 2017), among others. However, object detection in complex agricultural environments remains challenging due to issues such as occlusion, low detection accuracy, slow speed, large model parameters, and high computational complexity. For instance, in grape detection tasks, factors such as the dense arrangement of grape fruits and occlusion by tree leaves result in decreased accuracy of classical object detection models. To address the deficiencies of classical models in grape detection tasks, numerous researchers have refined classical algorithms to fulfill the demands for detection accuracy and real-time performance. Guo et al. (2023) replaced cross-stage partial networks (CSP) in the backbone of YOLOv4 with Resblock_body_AM, in which the output of each Resblock_body uses a simple, parameter-free attention module (SimAM) to refine features. Subsequently, they used bidirectional feature pyramid network (BiFPN) fusion weights to process the output of concatenate (Concat) and introduced skip connection structures to alleviate feature information loss. Additionally, they adjusted hyperparameters *α* and *γ* to 0.75 and 2 in the focal loss function to address the issue of imbalanced positive and negative samples. Experimental results demonstrated that YOLOv4+ achieved a 3.35% increase in mean average precision (mAP) and a 3% improvement in F1 compared to the original model. Li et al (Li et al., 2021. replaced LeakyReLU with Mish activation function to improve prediction accuracy, introduced squeeze and excitation (SE) attention to improving recognition ability, replaced convolution (Conv) in YOLOv4-tiny with depthwise separable convolution (DSC) to reduce model parameters and obtain real-time performance, and utilized soft nonmaximum suppression (Soft-NMS) to improve the model’s detection capability for overlapping grapes. Additionally, they applied transfer learning to enhance the model’s detection precision and generalization. When compared with Faster R-CNN, SSD300, YOLOv4, and YOLOv4-tiny, their proposed model achieved an increase in mAP of 1.67%, 2.28%, 0.84%, and 6.69%, respectively. Chen et al. (2023) replaced CBM (Conv+BN+Mish) with GBM (GhostConv+BN+Mish) in the backbone of YOLOv4, reducing the model’s parameters. They also integrated SE attention in residual blocks to focus on essential information. Furthermore, they added ASFF to the detection head to learn spatial weights for the fusion of features at different scales. Additionally, they constructed a new loss function to improve detection efficiency. Compared to the original model, their proposed model achieved a 3.69% increase in mAP, a 20.245 FPS improvement in detection speed, and a remarkable 82.79% reduction in parameters. Lu et al. (2022) captured long-distance dependencies, preserved both global and local features, and improved the detection accuracy and generalization ability by replacing the last C3 in the backbone of YOLOv5 with a Swin-transformer encoder block. The proposed Swin-T-YOLOv5 outperformed YOLOv5 in grape bunch detection, achieving a 4% higher mAP on cloudy days. This method could serve as a reliable digital tool to assist growers in performing precision management in vineyards. Zhu et al. (2023) incorporated convolutional block attention module (CBAM) attention at the end of the backbone of YOLOv5 to boost feature extraction. Additionally, a small object detection layer was added to preserve more information related to small objects. Furthermore, they replaced the original detection head with the decoupled head from YOLOX, where classification and regression are handled separately to optimize model performance. The results demonstrated that YOLOv5m-CFD achieved a 26.3% increase in mAP50–95 compared to YOLOv5m, making it well-suited for real-time grape harvesting.

From the above, it can be concluded that for automated equipment, maintaining real-time robustness and accuracy while preserving lightweight design is crucial. Therefore, the thesis designs a lightweight object detection model based on YOLOv8 for detecting grape bunches and evaluating the biophysical anomalies of grape bunches. The primary contributions of this thesis are as follows: Firstly, it incorporates modified efficient channel attention (MECA) to efficiently capture local cross-channel interactions and enhance feature expressiveness, thereby extracting crucial features. It retains more information on each channel without incurring a significant computational cost. Secondly, it utilizes a novel lightweight operator called group shuffle convolution (GSConv) to reconstruct bottleneck and C2f, creating an efficient feature fusion network slim-neck to replace neck in YOLOv8. This results in an object detection model with improved inference speed and reduced model parameters and computational complexity while maintaining precision. Thirdly, it proposes a new spatial pyramid pooling technique, a new spatial pyramid pooling fast (NSPPF), to replace SPPF to capture multiscale receptive field information for local and global feature fusion, promote channel information fusion, and enrich semantic information. Finally, it introduces a highly lightweight and effective dynamic upsampler, DySample, which redefines the upsampling process through point sampling. Compared to other upsamplers, DySample achieves excellent performance with minimal computation (lower inference latency, memory usage, and parameters), making it highly practical. Additionally, it adopts intersection over union with minimum points distance (MPDIoU) to replace complete intersection over union (CIoU) as a boundary box regression loss metric and adopts varifocal loss (VFL) as a classification loss metric. The MPDIoU loss uses the minimum point distance for bounding box similarity, directly minimizing the point-to-point distance between predicted and actual annotated bounding boxes. MPDIoU achieves faster convergence and more accurate regression results. The overall framework of the proposed method is illustrated in **Supplementary Figure S1**.

The remaining sections of the thesis include the following: Section 2 provides a brief introduction to the datasets used in the experiment and the proposed method. Section 3 outlines the hardware and software equipment, hyperparameters, and evaluation metrics used in the experiment. Section 4 discusses the experimental results. Section 5 presents the conclusions drawn from the experimental results.

## 2 Materials and methods

### 2.1 Selection of dataset

Pinheiro et al. (2023) collected two datasets: grapevine bunch detection and grapevine bunch condition detection. The datasets were obtained from the vineyard of the Faculty of Sciences at the University of Porto’s agrarian campus in Vairão. Both datasets use the same set of images and different labels; the “Bunch” is used to annotate the grape bunches in the image, and the condition of the grape bunches is distinguished using the “OptimalBunch” and “DamagedBunch”. These images come from red and white grapevine varieties and are collected under different lighting and perspective conditions, containing sufficient visual information. Additionally, some images have portions of the vine in addition to the target, and some images have scenes where different plant structures (i.e., trunks, leaves, stems, or other bunches) are occluded and bunches overlapped, adding complexity to the background environment. The purposes of these datasets are as follows: (1) The former is aimed at identifying grapevine bunches, which helps to utilize equipment to assist in harvesting. (2) The latter is used to classify the condition of grapevine bunches based on the presence of biophysical anomalies, defined as having 10% or more of any physical damage. The objective is to detect the condition of grapevine bunches, reduce yield losses, and assist vineyard managers in improving crop efficiency and quality. The images were taken using a Xiaomi Redmi Note 7 smartphone with a dual camera and a resolution of 8,000 pixels × 6,000 pixels. A total of 910 original images containing the target objects of grapevine bunches were collected. To reduce complexity, the resolution of these images is downscaled to 720 pixels × 540 pixels. Subsequently, the images undergo 10 different augmentation techniques, including rotation (rotating the image by + 15°, − 15°), scaling, translation (translating the image), flipping (mirroring the image horizontally), multiplying (making the image brighter or darker), blurring, adding noise (adding Gaussian noise), combination 1, and combination 3 (random combinations of three operations). The results of these augmentation operations are shown in **Supplementary Figure S2**. This results in two datasets, each containing 10,010 images. Each dataset was divided into three sets: Train (5995), Val (1980), and Test (2035). The details of the datasets are illustrated in **Table 1**.

**Table 1:** Number of images and annotated objects per class in each set after augmentation.

Dataset	Class	Images	Annotations			
			Train	Val	Test	Total
Grapevine bunch detection	Bunch	10,010	6,912	2,329	2,431	11,672
Grapevine bunch condition detection	OptimalBunch	7,678	4,958	1,637	1,826	8,421
	DamageBunch	3,045	1,954	692	605	3,251

In cases of limited data, data augmentation effectively expands the dataset, providing the model with more samples for training, which improves the training outcome. Data augmentation techniques such as rotation, scaling, and flipping simulate various possible real-world scenarios, making the model more robust to variations in the input data and thus enhancing its stability and reliability. By increasing the diversity and quantity of data, the model can learn the features of the data more comprehensively, resulting in higher prediction accuracy. Therefore, 10 augmentation operations were chosen, with each original image generating ten new versions of realistic vineyard images.

The thesis validates the effectiveness of the proposed modules and model on these datasets, offering a novel solution for automatically identifying grape bunches and classifying grape bunches as healthy or damaged. Applying the proposed modules or model can significantly improve the efficiency of managers in managing crops.

### 2.2 Overview of YOLOv8

**Figure 1:**
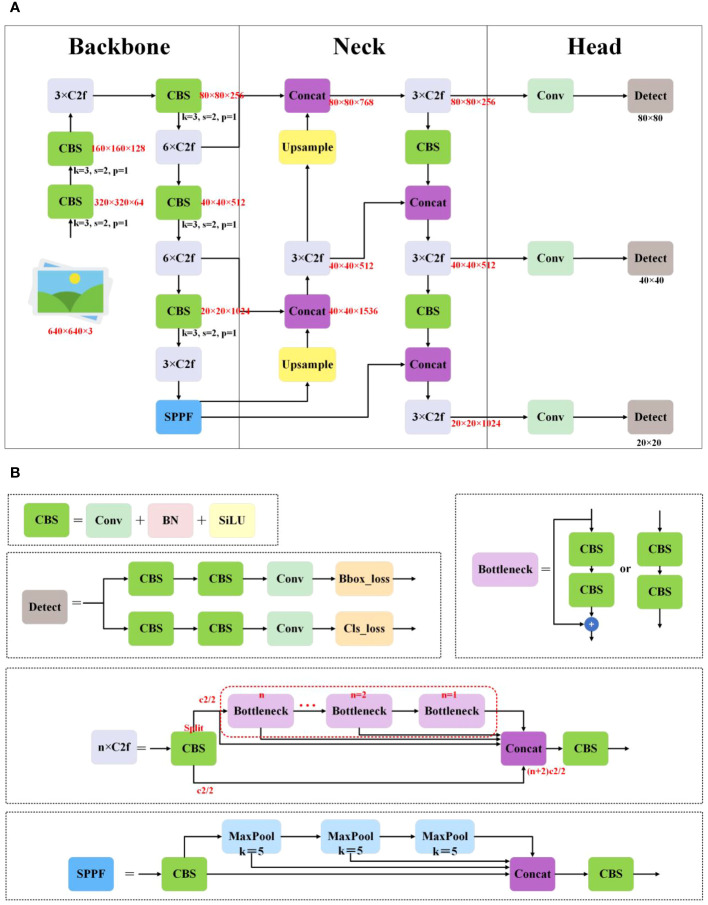
**(A)** The structure of YOLOv8. **(B)** The principal modules of YOLOv8.

YOLOv8 (Ultralytics, 2023) comprises five different versions: YOLOv8n, YOLOv8s, YOLOv8m, YOLOv8l, and YOLOv8x. Its network architecture, illustrated in **Figure 1A**, comprises a backbone, neck, and head, while its main modules, including CBS, Bottleneck, C2f, SPPF, and Detect, are depicted in **Figure 1B**. CBS, composed of Conv, batch normalization, and activation functions, aims to extract high-level semantic features from images for subsequent object detection tasks. The bottleneck module reduces channel dimensions using a 1 × 1 convolutional kernel, followed by processing feature maps with a 3 × 3 convolutional kernel. This design significantly reduces model complexity while maintaining strong feature representation capability. The C2f module consists of two Conv and multiple bottleneck blocks, utilizing residual connections to better learn and utilize correlated information between features. The SPPF module captures information from different receptive fields by applying maximum pooling operations to input feature maps at various receptive fields, then merging these pooled feature maps to provide a comprehensive spatial information representation. The Detect, which serves as YOLOv8’s detection head, predicts the positions and categories of objects in images. It achieves this by introducing convolutional and logistic regression operations in the final layers of the network to generate the positions of target boxes and corresponding class probabilities. YOLOv8 combines the above modules to achieve efficient object detection and recognition. It is the latest iteration of the YOLO series for object detection and image segmentation, developed by Ultralytics. Building upon the success of previous versions, it introduces new features and improvements to enhance performance and flexibility. Its key innovations and improvements are as follows:

1. Designing the C2f module, inspired by YOLOv5’s C3 module and YOLOv7’s ELAN, for further lightweighting while maintaining a richer gradient flow of information.
2. Replacing the conventional detection head of YOLOv5 with the decoupled head of YOLOX, separating the classification (cls) task and the regression (reg) task to expedite convergence.
3. Adopting the anchor-free concept, departing from the anchor-based approach used in previous versions.
4. Utilizing the VFL for classification loss, the distribution focal loss (DFL) and the CIoU for regression branch loss function ensures strong alignment between classification and regression tasks.
5. Adopting the task-aligned assigner matching method replaces the previous IoU matching or unilateral proportion allocation method.

### 2.3 Modified efficient channel attention

**Figure 2:**
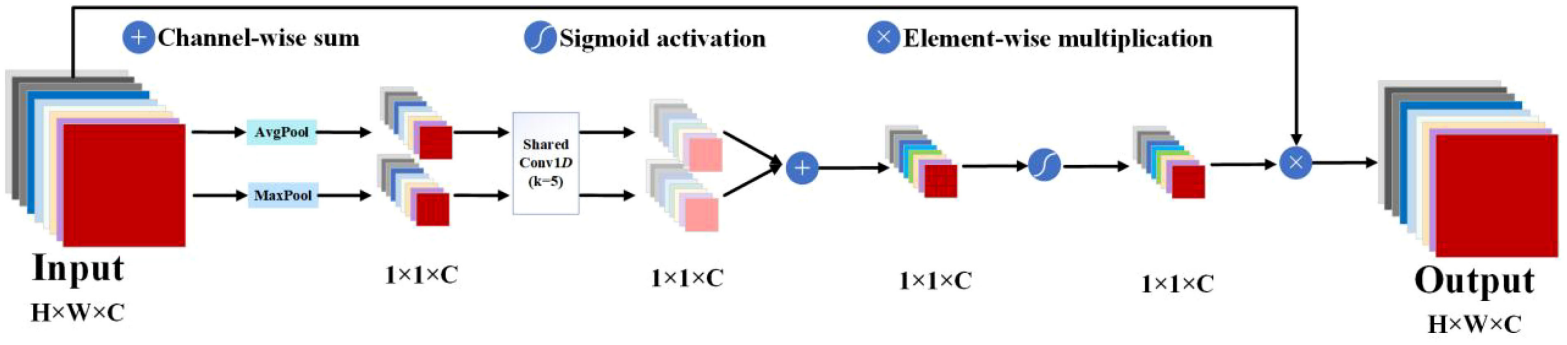
The structural diagram of MECA, “AvgPool” and “MaxPool”, represents average pooling and maximum pooling, respectively.

To enhance feature representation performance, network architectures have become increasingly complex, with deeper layers and a higher number of parameters (Xiao et al., 2020). While this allows models to learn richer features and improve their feature extraction and expression capabilities, it also leads to the stacking of more deep convolutional counterparts and significantly increased demands on memory and computational resources. Attention mechanisms offer a solution by not only strengthening the extraction of critical features and significantly improving performance (Brauwers and Frasincar, 2023) but also by being flexible in their integration at any point within the structure of CNNs. As a result, attention mechanisms have demonstrated substantial potential in computer vision (Guo et al., 2022); among them, the channel attention mechanism is employed to enhance the representation capability of each channel within CNNs. The fundamental idea is to weight the features of each channel, enabling the model to more effectively learn the correlations and significance among different channels. By modeling interdependencies among channels, the SE attention mechanism (Hu et al., 2018) enriches the discriminative capability of channel features, adaptively adjusts channel feature responses, mitigates the influence of irrelevant channels, and amplifies the importance of critical channels. The key operations encompass squeeze and excitation. Squeeze conducts global pooling to endow the model with a global receptive field. Excitation leverages the information from the squeeze operation to fully capture channel dependencies. SE effectively improves performance across diverse tasks, including classification, detection, and segmentation. However, dimensionality reduction has a side effect on prediction, and capturing dependencies across all channels is inefficient and unnecessary. The efficient channel attention (ECA) mechanism (Wang et al., 2020) avoids dimensionality reduction and effectively captures cross-channel interactions. Following global average pooling across channels without dimensionality reduction, ECA captures local cross-channel interactions by using one-dimensional convolutions to capture interactions between each channel and its k neighbors. This method has been proven to ensure efficiency and effectiveness. However, using only average pooling to aggregate spatial information has limitations. Guo et al. (2024) propose a new attention mechanism called MECA, which adds maximum pooling to the ECA mechanism. This structure aggregates information using both average pooling and maximum pooling, enabling the model to acquire more information about the target. Additionally, the parallel structure ensures detection speed. The structure of MECA is illustrated in **Figure 2**. Global average pooling and maximum pooling are employed to encapsulate global information and target salient feature information into a channel descriptor, respectively. Subsequently, 1-D convolution is employed to capture local cross-channel interactions for k neighbors of each channel to effectively learn channel attention. The obtained channel information is then aggregated via channel-wise summation. This is followed by the application of a sigmoid function to enhance non-linear expression capability. Ultimately, the key features are derived by multiplying the channel feature maps with the corresponding weight coefficients.

Shallow features play a crucial role in aiding the model’s understanding of detailed target information, such as contours, edges, colors, textures, corners, and shape features. Therefore, the thesis improves the backbone by integrating MECA, which boosts the backbone’s ability to extract shallow features, consequently improving the model’s detection precision.

### 2.4 Slim-neck

In DL, both Conv and DSC are crucial tools for feature extraction. Conv, being a fundamental operation, is typically utilized in image processing tasks. It extracts features by performing element-wise multiplication and summation on the input data (such as images) using a set of convolutional kernels (also known as filters). The convolution operation possesses characteristics such as parameter sharing and a local receptive field, enabling models to efficiently process large-scale input data while exhibiting a degree of translational invariance. However, traditional convolution requires a large number of parameter counts, especially when processing high-dimensional input data. As the input data size increases, the computational workload of the convolution operation also escalates, potentially resulting in performance degradation in resource-constrained environments. Lightweight designs can effectively alleviate the high computational costs associated with DL. Currently, the primary approach to reducing parameters and floating-point operations per second (FLOPs) involves the use of DSC, which improves processing speed. DSC comprises two steps: Depthwise Convolution (DWConv) and Pointwise Convolution (PWConv). In the DWConv step, each channel of the input data undergoes convolution with a separate kernel, generating multiple channel-wise feature maps. Subsequently, in the PWConv step, a 1 × 1 convolutional kernel is applied to each channel’s feature map to integrate information across channels. The primary advantage of DSC lies in its reduced parameter count and higher computational efficiency while still maintaining effective feature representation. This makes it particularly suitable for model designs in resource-constrained scenarios, such as mobile devices or embedded systems. However, the separation of depthwise convolution and pointwise convolution in DSC may hinder the model from fully capturing interchannel correlations. Therefore, in some cases, DSC might limit the model’s feature representation capability. In summary, while both Conv and DSC are essential feature extraction operations, they each possess limitations. The choice between them in practical applications depends on specific task requirements and computational resource constraints.

Achieving a balance between lightweight design and precision is of equal importance. Li et al. (2022) propose GSConv, as illustrated in **Supplementary Figure S3**. GSConv enhances nonlinear expressions and reduces redundancy or duplicated information by incorporating the DWConv and channel shuffle (CS) operations. It combines the advantages of Conv and DWConv, resulting in fewer parameters and efficient computing power while maintaining excellent feature expression ability. Additionally, it endeavors to preserve hidden connections between channels with lower time complexity.

**Figure 3:**
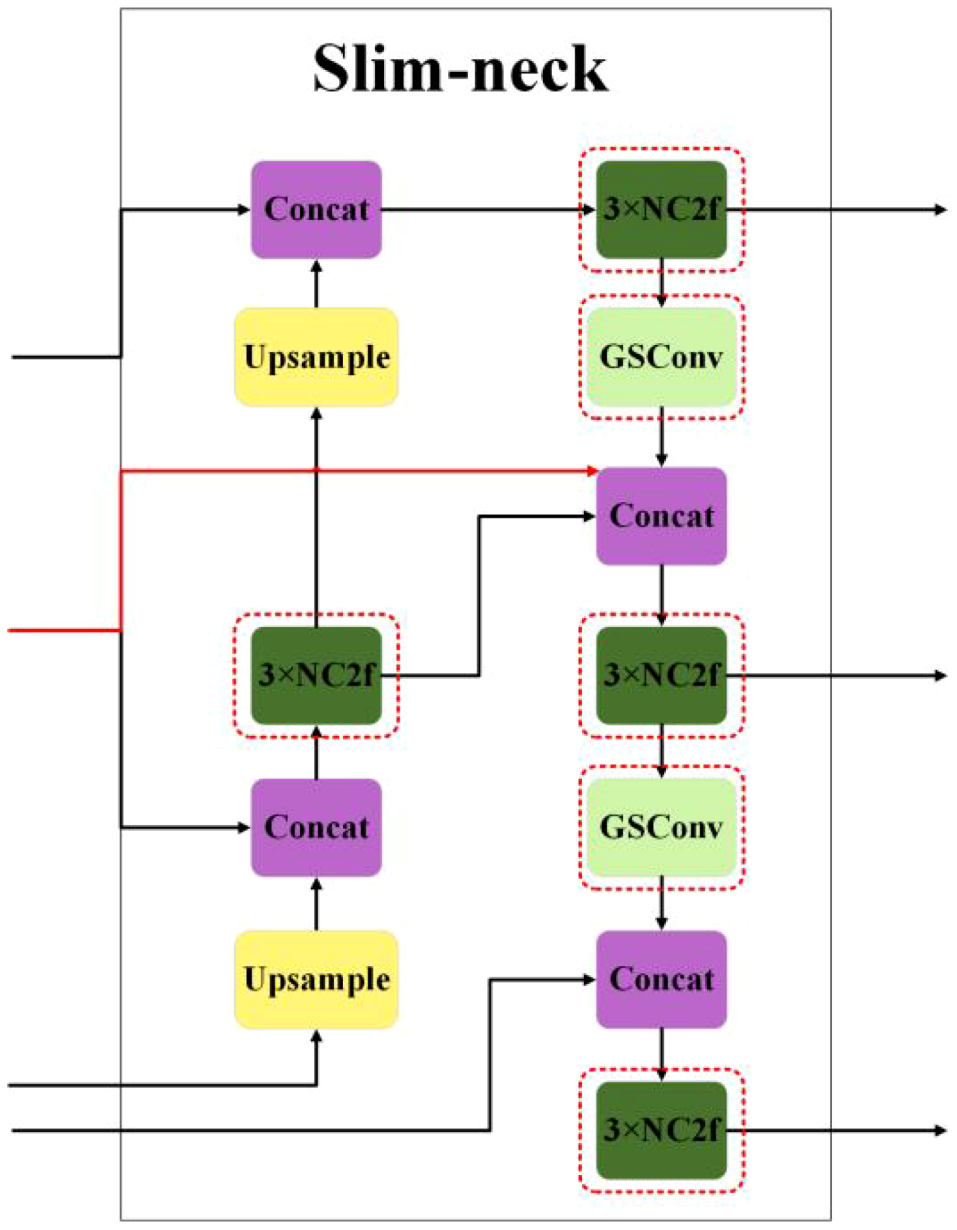
The structural diagram of slim-neck.

**Figure 4:**
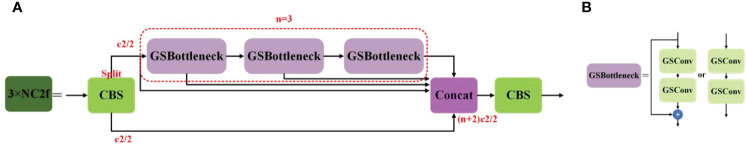
The structural diagrams of **(A)** the GSBottleneck module and **(B)** the NC2F module.

When the feature map reaches the neck network of the model, it already has the maximum number of channels and the minimum width and height dimensions, meaning it contains high-level semantic information. In this scenario, it may be considered to reduce the complexity of the neck. To reduce the loss of shallow feature information, drawing inspiration from DenseNet, the thesis introduces skip connections to mix shallow and deep-level features, achieving feature fusion for reusability and enhancing detection precision, and reconstructing the neck with both GSConv and NC2f. Next, it replaces the neck in YOLOv8 with a slim-neck, as depicted in **Figure 3**. The structure of GSBottleneck is shown in **Figure 4A**, and the structure of NC2f is shown in **Figure 4B**.

### 2.5 New spatial pyramid pooling fast

**Figure 5:**
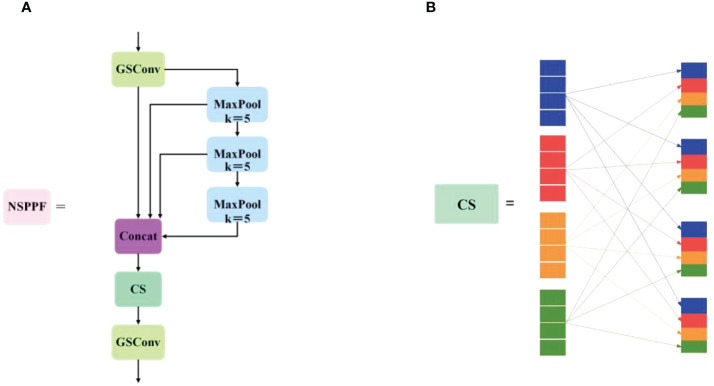
The structural diagrams of **(A)** the NSPPF module and **(B)** the channel shuffle module.

The receptive field, also known as the area that a convolutional neural network feature can see in an input image, plays a crucial role in object detection. A large receptive field captures global and high-level semantic features but may overlook small objects, resulting in poor detection of small targets. Conversely, a small receptive field gathers excessive local details and may miss the global context, affecting object recognition. Considering the multiscale nature of grape bunches and the need for model lightweightness, inspired by YOLOv5’s SPP (He et al., 2015), SPPF, feature fusion with the attentional multiple receptive fields (FFARF) (Qi et al., 2023), and GSConv, this thesis designs the NSPPF module, as shown in **Figure 5A**. Its specific improvements are as follows: (1) Inserting CS after the Concat module of SPPF facilitates interaction among channels with different receptive fields, enhancing intergroup communication and enriching target features. (2) Replacing the CBS module of SPPF with the GSConv operator reduces parameters and computations while maintaining speed, achieving equivalent detection performance. The CS module reorders channel sequences to facilitate better feature correlation capture, thereby enhancing the model’s performance and expressive capability, as shown in **Figure 5B**. To uniformly integrate channels from various receptive fields and augment cross-group communication, it is used to allocate each group with subgroups originating from diverse receptive fields and ensures equitable dispersion of channels among groups, facilitating effective interdependence capture across all receptive fields. Compared with SPP and SPPF, the NSPPF module serves the same purpose, has fewer parameters, and obtains richer feature information, thereby improving detection speed and precision.

### 2.6 Dynamic upsampler

**Figure 6:**
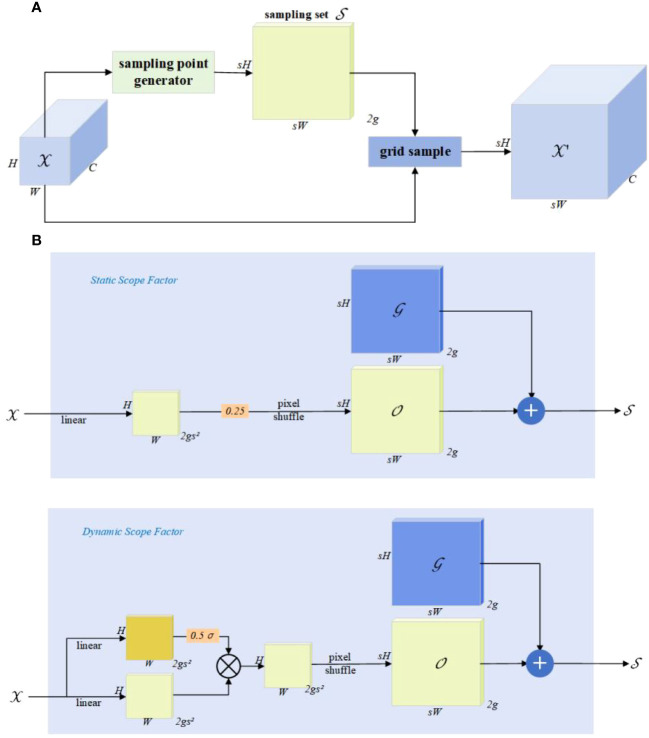
The design of DySample. The input feature, upsampled feature, generated offset, original grid, and sampling set are represented by X , $X^{\prime }$ , O , G , and S , respectively. **(A)** The sampling set S is generated by the sampling point generator, and the input features X are resampled using a grid sample ( grid_sample ), the calculation formula for this process is shown in Equation 1. In sampling point generator **(B)**, the sampling set S is the sum of the generated offset O and the original grid G , calculated as shown in Equation 2. It has two versions: static range factor and the dynamic range factor. The upper box displays the version with a “static range factor”, employing a linear layer for generating the offset, calculated as shown in Equation 3. The bottom one shows the version with a “dynamic range factor”, where the range factor is first generated and then used to modulate the offset, calculated as shown in Equation 4, where *σ* representation sigmoid function and *s* representation is the upsampling scale factor.


Feature upsampling is a crucial factor in progressively restoring feature resolution. In recent years, several upsamplers, such as content-aware reassembly of features (CARAFE) (Wang et al., 2019), fuse the assets of decoder and encoder (FADE) (Lu et al., 2022a), and similarity-aware point affiliation (SAPA) (Lu et al., 2022b), have contributed to improving performance. However, these methods, involving dynamic convolutions and extra subnetworks for generating dynamic kernels, are computationally intensive. Furthermore, FADE and SAPA are restricted to high-resolution images, limiting their applicability. To address these challenges, Liu et al. (2023) propose DySample, which takes a different approach to upsampling, bypassing dynamic convolutions by reframing upsampling through point sampling. DySample does not require high-resolution images, saving computational resources and achieving a lightweight design. Moreover, it can be effortlessly implemented using standard built-in functions in pytorch. The design of DySample is illustrated in **Figure 6**.

(1) $$X^{\prime }=ɡrid\_sample\left(X,S\right)$$

(2) S=G+O

(3) O=0.25linear(X)

(4) $$O=0.5\sigma \left(linear_{1}\left(X\right)\right)*linear_{2}\left(X\right)$$

Liu et al. (2023) use linear projection to generate point-wise offsets and to resample point values with the grid sample function in pytorch. They then progressively improve it by (i) controlling the initial sampling position, (ii) adjusting the moving scope of the offsets, and (iii) dividing the upsampling process into several independent groups and obtaining a new upsampler, DySample.

Compared to other dynamic upsamplers, DySample not only reports the best performance but also does not require high-resolution guided features as input. It does not need a custom CUDA package and consumes the least computational resources, showing advantages in terms of latency, training memory, training time, GFLOPs, and parameter count. For future work, DySample will be applied to low-level tasks and explore joint modeling of upsampling and downsampling. Therefore, the thesis combines DySample with the neck, achieving high practicality and rapid detection capability at low computational costs.

### 2.7 Intersection over union with minimum points distance

**Figure 7:**
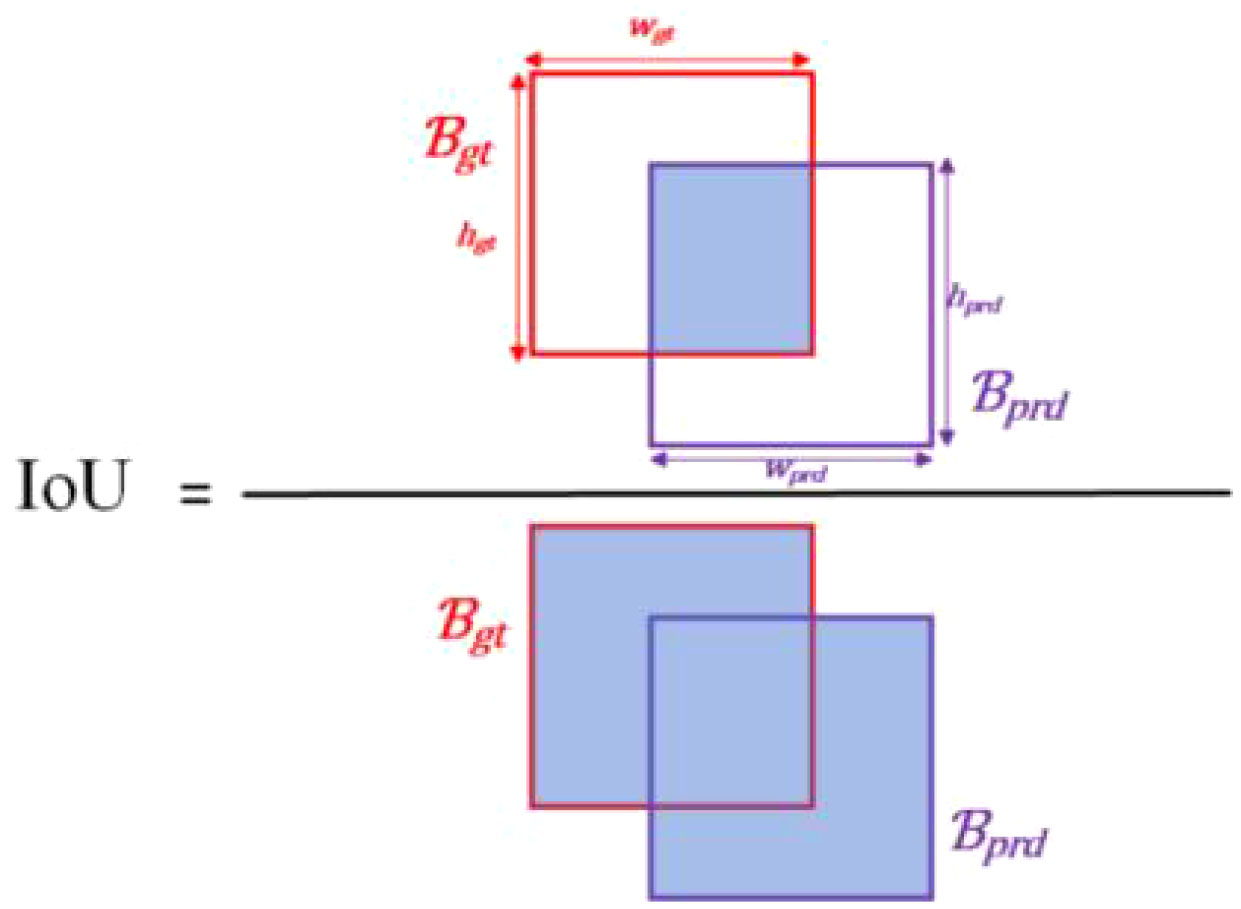
The diagram of IoU.


Intersection over union (IoU) (Yu et al., 2016) is the ratio of the union to the intersection of the ground truth box ${ℬ}_{ɡt}$ and predicted box ${ℬ}_{prd}$ , as illustrated in **Figure 7**. Where $h_{ɡt}$ , $w_{ɡt}$ is height and width of ${ℬ}_{ɡt}$ . $h_{prd}$ , $w_{prd}$ is height and width of ${ℬ}_{prd}$ . The calculation formula for IoU is shown in Equation 5. It is used to assess the similarity between the model’s detection result and the real target.

(5) $$IoU=\frac{{ℬ}_{ɡt}\cap^{}{ℬ}_{prd}}{{ℬ}_{ɡt}\cup^{}{ℬ}_{prd}}$$

YOLOv5 has three types of loss functions, which are box_loss (localization loss), obj_loss (confidence loss), and cls_loss (classification loss). YOLOv8 removes the obj_loss and uses VFL for classification loss. To align with the Anchor-Free approach and enhance generalization, it adds the DFL loss, employing CIoU (Zheng et al., 2020) + DFL (dfl_loss) as the loss function for the regression branch. This allows the network to quickly focus on the distribution of the target location and its vicinity. The calculation formula for CIoU is shown in Equations 6–8.


(6) $$CIoU=IoU-\left(\frac{\rho^{2}\left({ℬ}_{ɡt},{ℬ}_{prd}\right)}{C^{2}}+\alpha \nu \right)$$

(7) $$v=\frac{4}{\pi^{2}}{\left(arctan\frac{w_{ɡt}}{h_{ɡt}}-arctan\frac{w_{prd}}{h_{prd}}\right)}^{2}$$

(8) $$\alpha =\{\begin{array}{c} 0,\;\;IoU<0.5\\ \frac{\nu }{\left(1-IoU\right)+v},\;\;IoU\geq 0.5 \end{array}$$

where $\frac{\rho^{2}\left({ℬ}_{ɡt},{ℬ}_{prd}\right)}{C^{2}}$ denotes normalized central point distance and *υ* is aspect ratio. *ρ* is specified as Euclidean distance, and $\rho^{2}\left({ℬ}_{ɡt},{ℬ}_{prd}\right)$ is the Euclidean distance of central points of two boxes ${ℬ}_{ɡt}$ and ${ℬ}_{prd}$ . $C^{2}$ is the diagonal length of the smallest enclosing box covering two boxes ${ℬ}_{ɡt}$ and ${ℬ}_{prd}$ . *α* is a weight parameter.

Although existing methods have demonstrated some effectiveness, current bounding box regression loss functions cannot optimize scenarios where predicted boxes and actual annotated boxes have the same aspect ratio but significantly different width and height values. Therefore, Siliang and Yong (2023) propose MPDIoU as a bounding box regression loss function to compare the similarity between ${ℬ}_{ɡt}$ and ${ℬ}_{prd}$ . The MPDIoU simplifies the computation process for comparing the similarity between two bounding boxes and considers all relevant factors in existing loss functions, such as overlap areas, nonoverlap areas, center-point distances, and deviations in width and height. As a result, it leads to quicker convergence during training and more precise regression results. The calculation formula for MPDIoU is shown in Equations 9–11.


(9) $$MPDIoU=IoU-\left(\frac{d_{1}^{2}}{h^{2}+w^{2}}+\frac{d_{2}^{2}}{h^{2}+w^{2}}\right)$$

(10) $$d_{1}^{2}={\left(x_{1}^{prd}-x_{1}^{ɡt}\right)}^{2}+{\left(y_{1}^{prd}-y_{1}^{ɡt}\right)}^{2}$$

(11) $$d_{2}^{2}={\left(x_{2}^{prd}-x_{2}^{ɡt}\right)}^{2}+{\left(y_{2}^{prd}-y_{2}^{ɡt}\right)}^{2}\;$$

where *w*, *h* is width and height of input image. The top-left and bottom-right coordinates of ${ℬ}_{prd}$ are denoted as $\left(x_{1}^{prd},y_{1}^{prd},x_{2}^{prd},y_{2}^{prd}\right)$ , and the top-left and bottom-right coordinates of ${ℬ}_{ɡt}$ are denoted as $\left(x_{1}^{ɡt},y_{1}^{ɡt},x_{2}^{ɡt},y_{2}^{ɡt}\right)$ .

### 2.8 Proposed method

**Figure 8:**
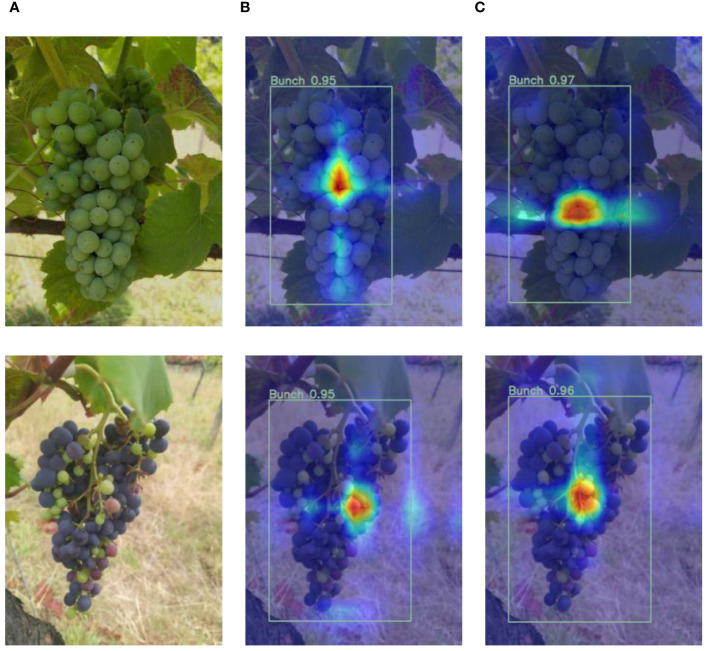
Visualization of the feature maps. **(A)** The original images; **(B)** the heatmaps of YOLOv8s; **(C)** the heatmaps of the YOLOv8s-grape.

To address issues related to low detection precision, slow speed, large model parameters, and computational demands, this thesis presents improvements to YOLOv8, proposing YOLOv8-grape, as illustrated in **Figure 8**. Firstly, the MECA module is introduced into the backbone, enriching channel information by combining different pooling layers and capturing local cross-channel interactions for k neighbors of each channel to effectively learn channel attention and obtain critical features. Following this, utilizing NSPPF replaces the original SPPF; its design combines the advantages of GSConv and CS to reduce redundancy and enhance the feature extraction capability of the model’s backbone. Subsequently, utilizing a slim-neck replaces the original neck; its design leverages the benefits of GSConv and skips connection to reuse shallow features, maintaining detection accuracy and speed while eliminating redundant functionalities. Finally, DySample is employed to replace Upsample, enhancing upsampling behavior at a low cost. Furthermore, MPDIoU is used as the loss function for the regression branch, improving the training of bounding box regression and thereby enhancing convergence speed and regression precision.

## 3 Experiment configuration

The hardware, running environment, configuration of CUDA, Cudnn, and related libraries for this experiment are detailed in **Table 2**. Model hyperparameters are presented in **Table 3**. Evaluation metrics are used to assess the overall model’s performance. In the field of ML, confusion matrices are often used to measure the accuracy of model classification in ML. For binary classification problems, the combination of real categories and the number of predicted categories by the model can be used, as shown in **Table 4**. In this experiment, we have selected parameters (Params) to measure the model’s training requirements in terms of volume. Giga floating-point operations per second (GFLOPs) are used to quantify the computational load of the model. Times (*T*) are used to measure the time of training for the model. P, R, mAP, and FPS are employed to validate the network’s performance. Further details are shown in **Table 5**.

**Table 2:** Experimental environment configuration.

System	CPU	GPU	CUDA	Cudnn	Pytorch
Windows 10	Intel(R) Core(TM) i9–9900K CPU @ 3.60 GHz	NVIDIA GeForce RTX 2080 Ti	10.1	7.6.5	1.8.1

**Table 3:** Model hyperparameter configuration.

Input image	Batch size	Epoch	Lr0	Lrf	Momentum	Weight decay
640 × 640	8	100	0.01	0.01	0.937	0.0005

**Table 4:** Binary confusion matrix.

Actual condition	Predicted condition	
	Positive	Negative
Positive	True positive (TP)	False negative (FN)
Negative	False positive (FP)	True negative (TN)

**Table 5:** The evaluation metrics.

Metrics	Abbreviation	Formula	Short description
Precision	*P*	$P=\frac{TP}{TP+FP}$	*P* is the proportion of the samples that the model predicted to be positive samples that are actually positive samples. *R* is the proportion of the actual positive samples and the model-predicted positive samples.
Recall	*R*	$R=\frac{TP}{TP+FN}$	
Average precision	AP	$AP=\int_{0}^{1}PR\;dR$	*AP* is the average precision of detecting one class.
Mean average precision	mAP	$mAP=\frac{\sum_{i=1}^{C}AP_{i}}{C}$	mAP is used to evaluate the performance of the model for all classes of mean average precision, where *C* is the total number of classes and *AP_i_* is the average precision for the *i*th class, where mAP50 represents the mean average precision for all classes when IoU = 0.5; mAP50–95 represents the mean average precision for all classes across various IoU thresholds, ranging from 0.5 to 0.95 in increments of 0.05.
Frames per second	FPS	$FPS=\frac{1000}{\left(t_{pre-process}+t_{inference}+t_{NMS}\right)}$	FPS is used to evaluate the speed of object detection, which is the number of images that can be processed per second or the time required to process one image to evaluate the detection speed.

TP refers to instances where the model predicts that an object is “Bunch” or a specific type of bunch, such as “OptimalBunch” or “DamageBunch”, and indeed, the object in the image belongs to the predicted class. Conversely, FP occurs when the model predicts that an object is a “Bunch” or a specific type of bunch, the object not belonging to the predicted class. FN denotes cases where the model predicts that an object is not a “Bunch” or a specific type of bunch, but the object actually belongs to the predicted class. TN signifies instances where the model predicts that an object is not a “Bunch” or a specific type of bunch, and indeed, the object in the image does not belong to the predicted class.

The smaller the parameters and GFLOPs, the easier it is to deploy the model on the mobile terminal of the picking robot; a higher FPS indicates shorter processing time and faster speed; a higher mAP reflects better performance.

## 4 Results and discussion

### 4.1 Results

To validate the proposed method, the thesis uses publicly available datasets, grapevine bunch detection, and grapevine bunch condition detection. The choice of baseline model for the experiment is presented in **Supplementary Table S1**. To verify the effectiveness of the proposed modules, ablation experiments are conducted as outlined in **Table 6**, where A, B, C, D, and E correspond to MECA, slim-neck, NSPPF, DySample, and MPDIoU, respectively. Additionally, in order to further verify the effectiveness of MECA, DySample, and MPDIoU, comparisons are made with other attention mechanisms [SE, ECA, normalization-based attention module [NAM] (Liu et al., 2021), efficient multiscale attention [EMA] (Ouyang et al., 2023)], upsampling methods (CARAFE) and loss functions [focal efficient intersection over union [Focal EIoU] (Zhang et al., 2022), and wise intersection over union [WIoUv3] (Tong et al., 2023)], as shown in **Tables 7**–**9**. In order to further verify the performance of YOLOv8s-grape, the proposed model is compared with YOLOv5s, YOLOv5m (Ultralytics, 2020), YOLOv6n, YOLOv6s (Li et al., 2022), Gold-YOLO-N, Gold-YOLO-S (Wang et al., 2023), YOLOv7-tiny, YOLOv7 (Wang et al., 2022), YOLOX-s (Ge et al., 2021), PP-YOLOE-s, PP-YOLOE-m (Xu et al., 2022), DAMO-YOLO-T, DAMO-YOLO-S (Xu et al., 2022), and YOLOv8s. The results are shown in **Table 10**. The validation results of the proposed method and baseline model at different IoU thresholds for mAP are shown in **Supplementary Table S2**.

**Table 6:** Ablation experiment.

Datasets	Baseline	A	B	C	D	E	Params (M)	GFLOPs	*P* (%)	*R* (%)	mAP50 (%)	mAP50–95 (%)	FPS
Grapevine bunch detection	✓						11.1	28.6	94.7	90.4	95.2	80.5	107.5
	✓	✓					11.1	28.7	93.9	90.8	95.9	81.2	98.0
	✓		✓				9.9	26.6	94.5	89.5	95.8	82.0	96.2
	✓			✓			10.8	28.4	95.5	90.0	95.9	81.0	106.4
	✓				✓		11.2	28.7	94.6	91.1	95.3	81.7	98.0
	✓					✓	11.1	28.6	94.8	90.6	95.1	81.0	107.5
Grapevine bunch condition detection	✓						11.1	28.6	89.2	87.3	90.1	76.0	106.4
	✓	✓					11.1	28.7	90.4	86.4	90.6	76.9	97.1
	✓		✓				9.9	26.6	89.6	86.9	90.9	77.7	94.3
	✓			✓			10.8	28.4	91.3	86.0	90.4	76.7	105.3
	✓				✓		11.2	28.7	90.8	85.1	90.4	76.8	96.2
	✓					✓	11.1	28.6	89.8	86.1	90.1	76.7	106.4

**Table 7:** Comparison of different attention mechanisms.

Method	Params (M)	GFLOPs	Grapevine bunch detection					Grapevine bunch condition detection				
			*P* (%)	*R* (%)	mAP50 (%)	mAP50–95 (%)	FPS	*P* (%)	*R* (%)	mAP50 (%)	mAP50–95 (%)	FPS
Baseline	11.1	28.6	94.7	90.4	95.2	80.5	107.5	89.2	87.3	90.1	76.0	106.4
SE	11.2	28.7	95.8	89.9	95.2	80.7	97.0	90.1	86.1	89.4	75.6	99.0
ECA	11.2	28.7	94.6	90.9	95.2	81.3	99.0	90.4	87	89.9	75.4	99.0
NAM	11.1	28.7	94	90.2	95.1	80.7	98.0	90.2	87.3	90.6	76.1	98.0
EMA	11.1	28.7	95.8	88.7	94.7	80.4	83.3	92	85	90.4	76.3	84.0
MECA	11.1	28.7	95.2	90.8	**96**	**81.3**	96.2	90.4	86.4	**90.6**	**76.9**	97.1

The bold values highlight that the proposed modules and models perform better compared to other benchmark methods.

**Table 8:** Comparison of different upsamplers.

Method	Params (M)	GFLOPs	Grapevine bunch detection					Grapevine bunch condition detection				
			*P* (%)	*R* (%)	mAP50 (%)	mAP50–95 (%)	FPS	*P* (%)	*R* (%)	mAP50 (%)	mAP50–95 (%)	FPS
Baseline (upsample)	11.1	28.6	94.7	90.4	95.2	80.5	107.5	89.2	87.3	90.1	76.0	106.4
CARAFE	11.2	28.8	93.9	90.3	95.3	80.7	67.1	90.9	85.5	88.8	75.5	66.2
DySample	11.2	28.7	94.6	91.1	**95.3**	**81.7**	98.0	90.8	85.1	**90.4**	**76.8**	96.2

The bold values highlight that the proposed modules and models perform better compared to other benchmark methods.

**Table 9:** Comparison of different loss functions.

Method	Grapevine bunch detection					Grapevine bunch condition detection				
	*P* (%)	*R* (%)	mAP50 (%)	mAP50–95 (%)	FPS	*P* (%)	*R* (%)	mAP50 (%)	mAP50–95 (%)	FPS
Baseline (CIoU)	94.7	90.4	95.2	80.5	107.5	89.2	87.3	90.1	76.0	106.4
Foca EIoU	93.5	90	95.5	80.7	107.5	89.5	86.2	89.3	75.7	105.2
WIoUv3	94.8	90.2	95.4	80.2	108.7	89.4	85.6	89.3	75.5	105.2
MPDIoU	94.8	90.6	**95.1**	**81**	107.5	89.8	86.1	**90.1**	**76.7**	106.4

The bold values highlight that the proposed modules and models perform better compared to other benchmark methods.

**Table 10:** Comparison of different methods.

Method	Params (M)	GFLOPs	Grapevine bunch detection					Grapevine bunch condition detection				
			*P* (%)	*R* (%)	mAP50 (%)	mAP50–95 (%)	*T* (h)	*P* (%)	*R* (%)	mAP50 (%)	mAP50–95 (%)	*T* (h)
YOLOv5s	7.0	16.0	94.4	91.3	94.6	75.6	2.3	90.1	87.0	89.1	69.5	2.4
YOLOv5m	20.9	48.2	95.3	90.4	95.7	78.8	3.6	89.6	88.0	89.3	72.7	3.6
YOLOv6n	4.6	11.4	–	–	94.8	75.2	2.8	–	–	90.0	71.4	2.9
YOLOv6s	18.5	45.3	–	–	93.8	76.1	3.4	–	–	89.4	72.6	3.4
Gold-YOLO-N	5.6	12.1	–	–	95.0	75.0	3.2	–	–	88.9	70.6	3.1
Gold-YOLO-S	21.5	46.0	–	–	94.1	75.3	3.7	–	–	88.7	70.7	3.7
YOLOv7-tiny	6.0	13.2	90.8	90.7	93.8	64.6	3.3	87.1	86.2	86.6	60.1	3.3
YOLOv7	37.2	105.1	93.0	90.7	95.4	74.8	6.4	88.2	84.6	87.9	68.2	6.4
YOLOX-s	8.9	26.8	–	–	93.6	61.9	5.0	–	–	87.1	58.0	4.7
PP-YOLOE-s	7.6	16.4	–	–	95.9	77.3	4.4	–	–	86.2	69.8	4.4
PP-YOLOE-m	23.4	49.6	–	–	95.7	78.6	20.1	–	–	86.3	70.5	23.4
DAMO-YOLO-T	8.6	18.2	–	–	95.0	73.3	6.0	–	–	89.5	68.8	6.0
DAMO-YOLO-S	16.3	38.0	–	–	94.0	72.8	9.8	–	–	90.0	70.2	9.8
YOLOv8s	11.1	28.6	94.7	90.4	95.2	80.5	2.3	89.2	87.3	90.1	76.0	2.3
YOLOv8s-grape	9.6	26.3	94.7	90.3	**95.5**	**82.4**	2.7	91.4	86	**91.4**	**78.6**	2.7

The bold values highlight that the proposed modules and models perform better compared to other benchmark methods.

As shown in **Supplementary Table S1**, Params, GFLOPs, P, R, mAP50, and mAP50–95 increase with the depths and widths of the model, while FPS decreases gradually. Specifically, on the grapevine bunch detection dataset, YOLOv8s shows an improvement of 1.9% in mAP50–95 compared to YOLOv8n, while YOLOv8m, YOLOv8l, and YOLOv8x exhibit increases of 1.6%, 2%, and 0.8% in mAP50–95 relative to YOLOv8s, respectively. On the grapevine bunch condition detection dataset, YOLOv8s demonstrates a 3.4% increase in mAP50–95 compared to YOLOv8n, while YOLOv8m, YOLOv8l, and YOLOv8x show improvements of 1%, 1.2%, and 1.8% in mAP50–95 relative to YOLOv8s, respectively. However, YOLOv8m, YOLOv8l, and YOLOv8x have parameter increases of 14.8 M, 32.5 M, and 57.1 M, and GFLOPs increases of 50.5, 136.8, and 229.5, respectively, compared to YOLOv8s. Obviously, this is not applicable in device-constrained scenarios. Therefore, the conclusion can be drawn that selecting the appropriate model depth and width can enhance detection performance while conserving computational resources. Hence, YOLOv8s is chosen as the baseline model due to its superior detection performance, fast detection speed, and compact model size. An improvement upon this baseline model will provide technical support for subsequent mobile deployments.

As shown in **Table 6**, the improvement points (MECA, slim-neck, NSPPF, DySample, and MPDIoU) yield varying degrees of enhancement. Specifically, on the grapevine bunch detection dataset, the mAP50–95 of models with these improvement points increased by 0.7%, 1.5%, 0.5%, 1.2%, and 0.5% compared to YOLOv8s, respectively. On the grapevine bunch condition detection dataset, these models exhibit mAP50–95 increases of 0.9%, 1.7%, 0.7%, 0.8%, and 0.7% compared to YOLOv8s, respectively. Their FPS remains largely consistent with the baseline model. Compared to YOLOv8s, the parameters and GFLOPs of slim-neck and NSPPF decrease by 1.2 million parameters and 2 Giga floating-point operations per second, and 0.3 million parameters and 0.2 Giga floating-point operations per second, respectively. Among these improvements, attention mechanisms, upsampling techniques, and loss functions enhance detection accuracy at a lower cost. The lightweight design of the proposed method is achieved through slim-neck and NSPPF. Slim-neck reduces the model’s parameter count and computational complexity by employing a meticulously designed lightweight network architecture. It incorporates GSConv, NC2f, and skip connections to reduce the model’s parameter count and computational overhead while maintaining detection performance. NSPPF employs the strategies of GSConv and CS to diminish the model’s parameter count, facilitating increased intragroup channel interaction and thereby accomplishing the model’s lightweight design. Therefore, the improvement points possess flexible and lightweight characteristics, enabling easy integration into various computer vision tasks, significantly enhancing feature representation capabilities, and achieving optimal performance.

As shown in **Tables 7** **–** **9**, it is evident that MECA, DySample, and MPDIoU consistently yield higher mAP values compared to other attentions, upsamplers, and loss functions, with minimal impact on speed. Specifically, on the grapevine bunch detection dataset, MECA exhibits mAP50–95 increases of 0.8%, 0.6%, 0.6%, and 0.9% compared to the baseline model, SE, NAM, and EMA, respectively. DySample demonstrates mAP50–95 increases of 1.2% and 1% compared to the baseline model and CARAFE, respectively. MPDIoU shows mAP50–95 increases of 0.5%, 0.3%, and 0.8% compared to the baseline model, Focal EIoU, and WIoUv3, respectively. On the grapevine bunch condition detection dataset, MECA achieves mAP50–95 increases of 0.9%, 1.3%, 1.5%, 0.8%, and 0.6% compared to the baseline model, SE, ECA, NAM, and EMA, respectively. DySample achieves mAP50–95 increases of 0.8% and 1.3% compared to the baseline model and CARAFE, respectively. MPDIoU achieves mAP50–95 increases of 0.7%, 1%, and 1.2% compared to the baseline model, Focal EIoU, and WIoUv3, respectively. From these conclusions, it is evident that the improvement points are more effective on these two datasets compared to other enhancement methods. Therefore, this thesis selects MECA, DySample, and MPDIoU for further exploration and implementation.

As shown in **Table 10**, compared with YOLOv5s, YOLOv5m, YOLOv6n, YOLOv6s, Gold-YOLO-N, Gold-YOLO-S, YOLOv7-tiny, YOLOv7, YOLOX-s, PP-YOLOE-s, PP-YOLOE-m, DAMO-YOLO-T, DAMO-YOLO-S, and YOLOv8s, the mAP50–95 of the YOLOv8s-grape is respectively higher by 6.8%, 3.6%, 7.2%, 6.3%, 7.4%, 7.1%, 17.8%, 7.6%, 20.5%, 5.1%, 3.8%, 9.1%, 9.6%, and 1.9% on the grapevine bunch detection dataset. The mAP50–95 of the YOLOv8s-grape is respectively higher by 9.1%, 5.9%, 7.2%, 6%, 8%, 7.9%, 18.5%, 10.4%, 20.6%, 8.8%, 8.1%, 9.8%, 8.4%, and 2.6% on the grapevine bunch condition detection dataset. Compared with YOLOv5s, YOLOv6n, YOLOv6s, Gold-YOLO-N, Gold-YOLO-S, YOLOv7-tiny, YOLOv7, YOLOX-s, DAMO-YOLO-T, DAMO-YOLO-S, and YOLOv8s, the mAP50 of the YOLOv8s-grape is respectively higher by 0.9%, 0.7%, 1.7%, 0.5%, 1.4%, 1.7%, 0.1%, 1.9%, 0.5%, 1.5%, and 0.3% on the grapevine bunch detection dataset. The mAP50 of the YOLOv8s-grape is respectively higher by 2.3%, 1.4%, 2%, 2.5%, 2.7%, 4.8%, 3.5%, 4.3%, 1.9%, 1.4%, and 1.3% on the grapevine bunch condition detection dataset. Compared to YOLOv5m, PP-YOLOE-s, and PP-YOLOE-m, the mAP50 of YOLOv8s-grape is respectively lower by 0.2%, 0.4%, and 0.2% on the grapevine bunch detection dataset. However, the T of YOLOv8s-grape is lower than theirs. Additionally, the mAP50 of YOLOv8s-grape is respectively higher by 2.1%, 5.2%, and 5.1% on the grapevine bunch condition detection dataset. Compared to YOLOv8s, the parameters and GFLOPs of the proposed method decrease by 1.5 million parameters and 2.3 Giga floating-point operations per second. Therefore, considering the comprehensive data, the proposed method has superiority over other methods. Specifically, the proposed method has relatively small parameters and computational requirements, while achieving high detection precision.

As shown in **Supplementary Table S2**, the proposed method shows an improvement in thresholds for different IoUs, indicating that the proposed method is effective in grapevine bunch detection and grapevine bunch condition detection.

### 4.2 Visualization

#### 4.2.1 Comparison of heatmaps of YOLOv8s and the proposed method

**Figure 9:**
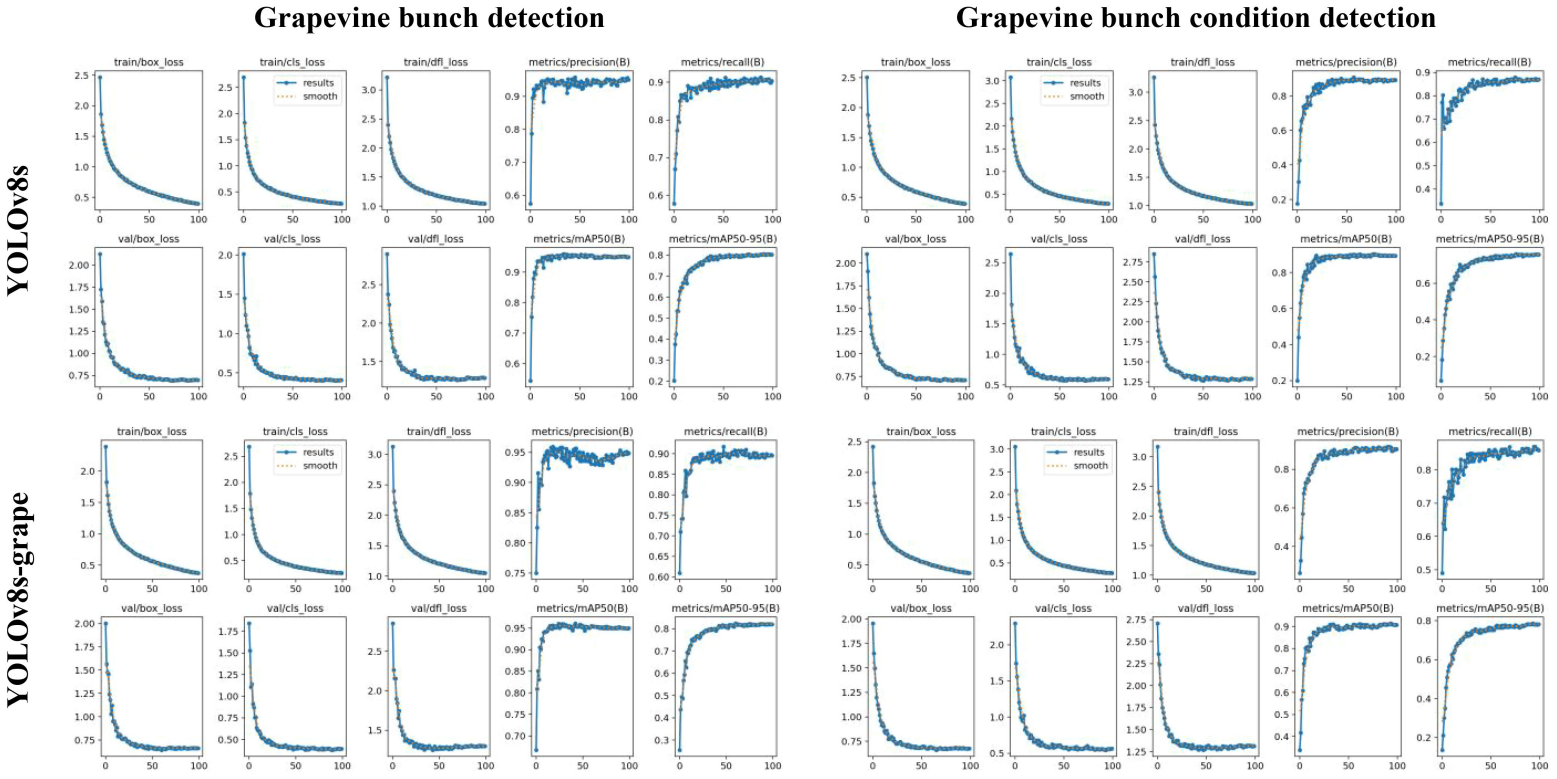
The box_loss, cls_loss, dfl_loss, P, R, mAP50, and mAP50–95 curves of the training and validating process of YOLOv8s and the proposed method. Where the x-axis is epochs, and the y-axis is the curve name.

To demonstrate the proposed method’s feature extraction capabilities more intuitively, this thesis uses Grad-CAM to visualize the feature map before entering the detection head of YOLOv8s and the proposed method. The results of the heatmaps visualized by the feature maps are shown in **Figure 9**, where red areas indicate the regions on which the model is highly focused.

It can be seen from the figure that, compared to YOLOv8s, the YOLOv8s-grape pays more attention to the areas of the grape bunch in the feature extraction process and relatively less attention to irrelevant information. Thus, it showed the proposed method can better focus on the important information of grape bunches and biophysical anomaly assessment.

#### 4.2.2 Comparison of results of YOLOv8s and the proposed method

To validate the performance of the proposed model, this thesis visualizes the comparison results between the proposed method and baseline model on two datasets, as shown in **Figure 9**.

From the graph, it can be seen that the descent curve of the loss function of the proposed method during the validation process is faster, indicating that the improved loss function helps to accelerate convergence. During the training process, when the mAP50 and mAP50–95 of the proposed method tend to stabilize, they are higher than YOLOv8s, indicating that the proposed method can improve overall detection performance.

### 4.3 Discussion

Agricultural automation (grape bunch detection, biophysical anomaly assessment) has always been a focal point in smart agriculture. Grape bunches can often be partially obscured by leaves or other parts of the grapevine, making accurate detection a challenging task. Furthermore, varying weather conditions can lead to differences in lighting, posing challenges for accurate grape detection under changing illumination. Especially with DL algorithms emerging as the mainstream research approach for vision systems in automated robots, there is a demand for model lightweighting to facilitate deployment on mobile devices. Grapevine bunch detection and biophysical anomaly assessment research should further enhance the real-time, precision, and reliability of grape detection, thus promoting widespread applications in agricultural automation machines. To address these challenges, the thesis optimizes YOLOv8 by integrating slim-neck and NSPPF to reduce model parameters, introducing attention mechanisms to enhance feature extraction capabilities, refining upsampling for improved practicality and rapid detection, and enhancing the loss function for faster convergence and more accurate regression results. Through the experiments outlined in Section 4, it was found that the proposed method, without significantly increasing Params and GFLOPs, improved detection performance. Lightweight models have fewer parameters and lower complexity, performing well in efficiency and resource utilization, but may limit their ability to capture complex patterns and relationships in data, leading to reduced predictive performance, especially for complex tasks or datasets with high variability. From the experimental data, although the mAP has increased and the detection performance has improved, the training time has also increased. The test results of YOLOv8s and the proposed method (YOLOv8s-grape) are shown in **Supplementary Figure S4**.

From **Supplementary Figure S4**, it can be seen that the proposed method can improve the precision of the model’s prediction of targets. This method can also be applied to other crops in the same growth state (clusters), such as tomatoes, bananas, and peppers.

## 5 Conclusion

The lightweight models play a pivotal role in advancing agricultural automation and sustainability. By reducing computational complexity and memory requirements, lightweight models enable efficient execution on devices with limited processing power, such as edge devices or mobile platforms. This is especially crucial for applications like real-time grape bunch detection and biophysical anomaly assessment in agricultural settings, where timely decision-making is essential for optimizing crop management and resource allocation. The thesis proposes a lightweight and efficient model for grape bunch detection and biophysical anomaly assessment in complex environments based on YOLOv8 by redesigning the network structure. Attention mechanisms have been added to help the model focus on important features. This enhancement can improve the model’s capability to detect obstructed or closely arranged grape clusters by highlighting the most critical areas in the images. The application of the slim-neck contributes significantly to the speed of grape detection, which is crucial for real-time automated detection and anomaly assessment. It reduces computational complexity while maintaining sufficient detection precision. The fusion of shallow and deep features aids the model in reducing the loss of object information, which is beneficial for grape bunch detection and biophysical anomaly assessment. The proposed NSPPF reduces the parameter and computational load while outperforming SPPF. The CS operation encourages cross-interactions among feature maps from different channels, enhancing the model’s understanding of relationships between various features. This helps improve the model’s ability to learn complex patterns and abstract features, thus enhancing its robustness. Optimizing upsampling aids in increasing resolution, information recovery, enhancing the performance of DL tasks, and improving image quality. Additionally, optimizing the loss functions enables the model to more accurately locate dense grape bunches. Compared to other methods, the proposed method exhibits superior precision, better generalization, and increased robustness. This thesis provides a theoretical foundation for grape bunch detection and biophysical anomaly assessment, further facilitating automation. It can also offer technical support for device deployment and serve as a reliable digital tool for providing accurate diagnoses to assist growers in taking timely actions to protect grapes, thereby improving work efficiency and reducing labor and computing resource costs. The proposed method and module design concepts can be incorporated into mobile devices or robotic systems, enabling real-time and precise grape management for agricultural practitioners in the future.

## Data availability statement

The data presented in this study are openly available in the GitHub repository: https://github.com/joiy123/v8-grape.

## Author contributions

WY: Conceptualization, Data curation, Formal analysis, Funding acquisition, Resources, Supervision, Validation, Writing – original draft, Writing – review & editing. XQ: Conceptualization, Data curation, Formal analysis, Investigation, Methodology, Project administration, Resources, Software, Supervision, Validation, Visualization, Writing – original draft, Writing – review & editing.
