# Supplementary figures and images for: A lightweight and efficient model for grape bunch detection and biophysical anomaly assessment in complex environments based on YOLOv8s
Source: Front Plant Sci. 2024 Aug 6;15:1395796. doi: 10.3389/fpls.2024.1395796 (PMC11333308; doi:10.3389/fpls.2024.1395796)

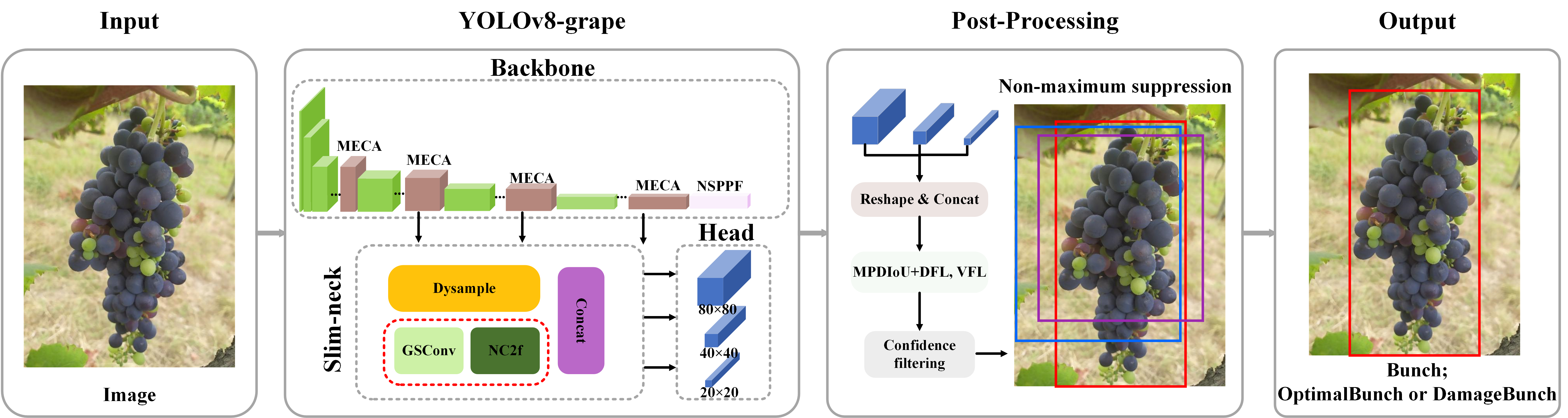

Supplement: Supplementary file 1 [file DataSheet_1.zip › New Data Sheet 1/Images/Supplementary Figure S1. The overall framework of the proposed method.tif]

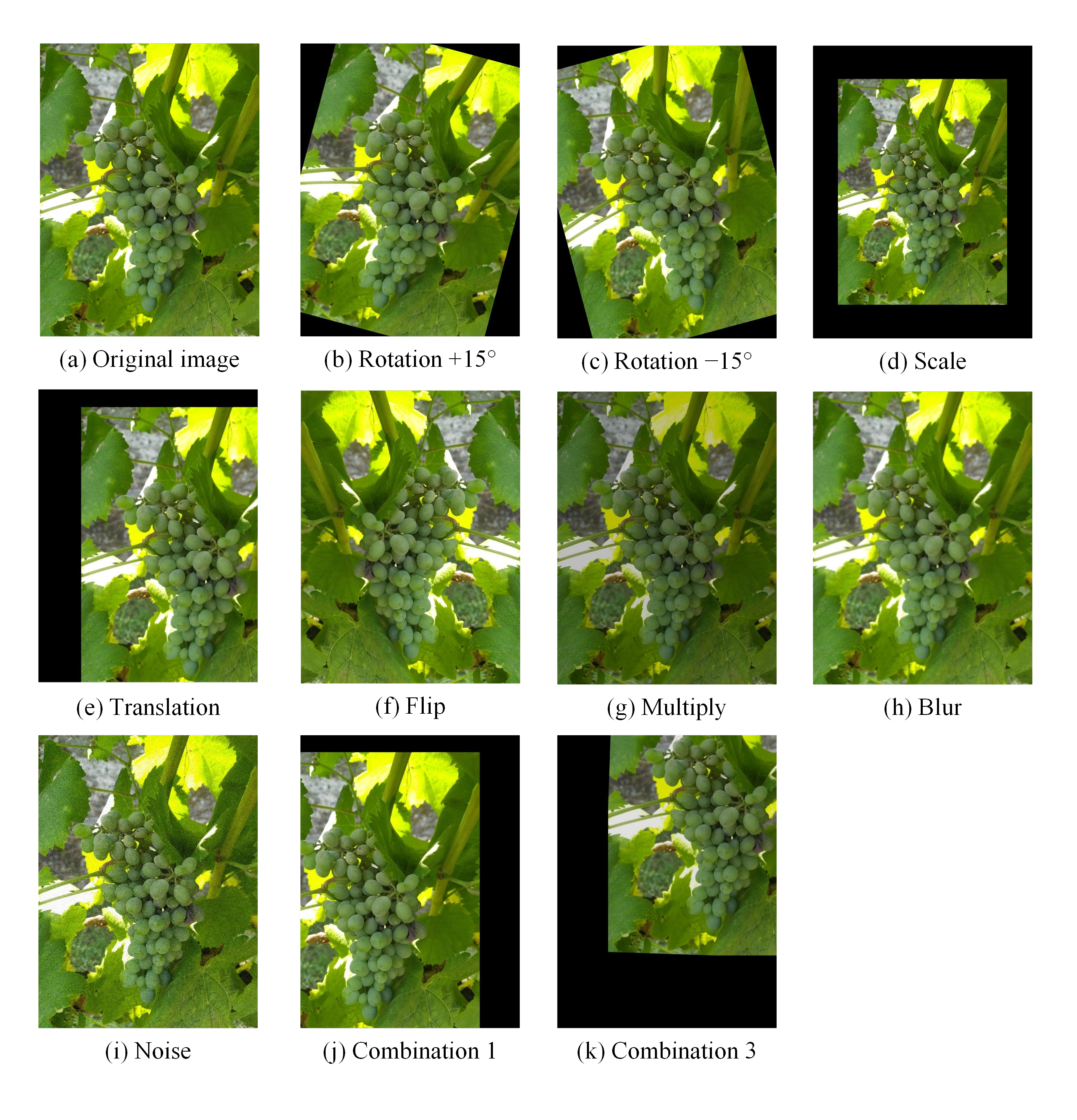

Supplement: Supplementary file 1 [file DataSheet_1.zip › New Data Sheet 1/Images/Supplementary Figure S2. Augmentation operations applied to datasets.tif]

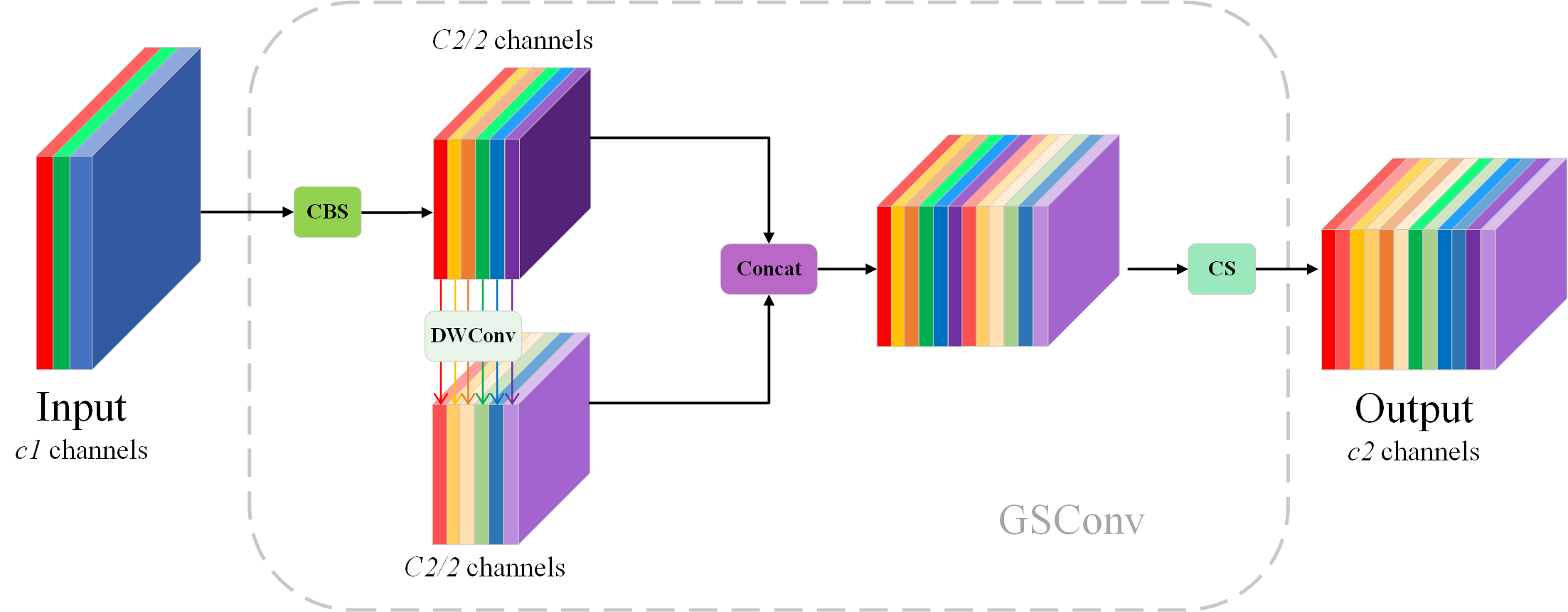

Supplement: Supplementary file 1 [file DataSheet_1.zip › New Data Sheet 1/Images/Supplementary Figure S3. The structural diagram of GSConv module.tif]

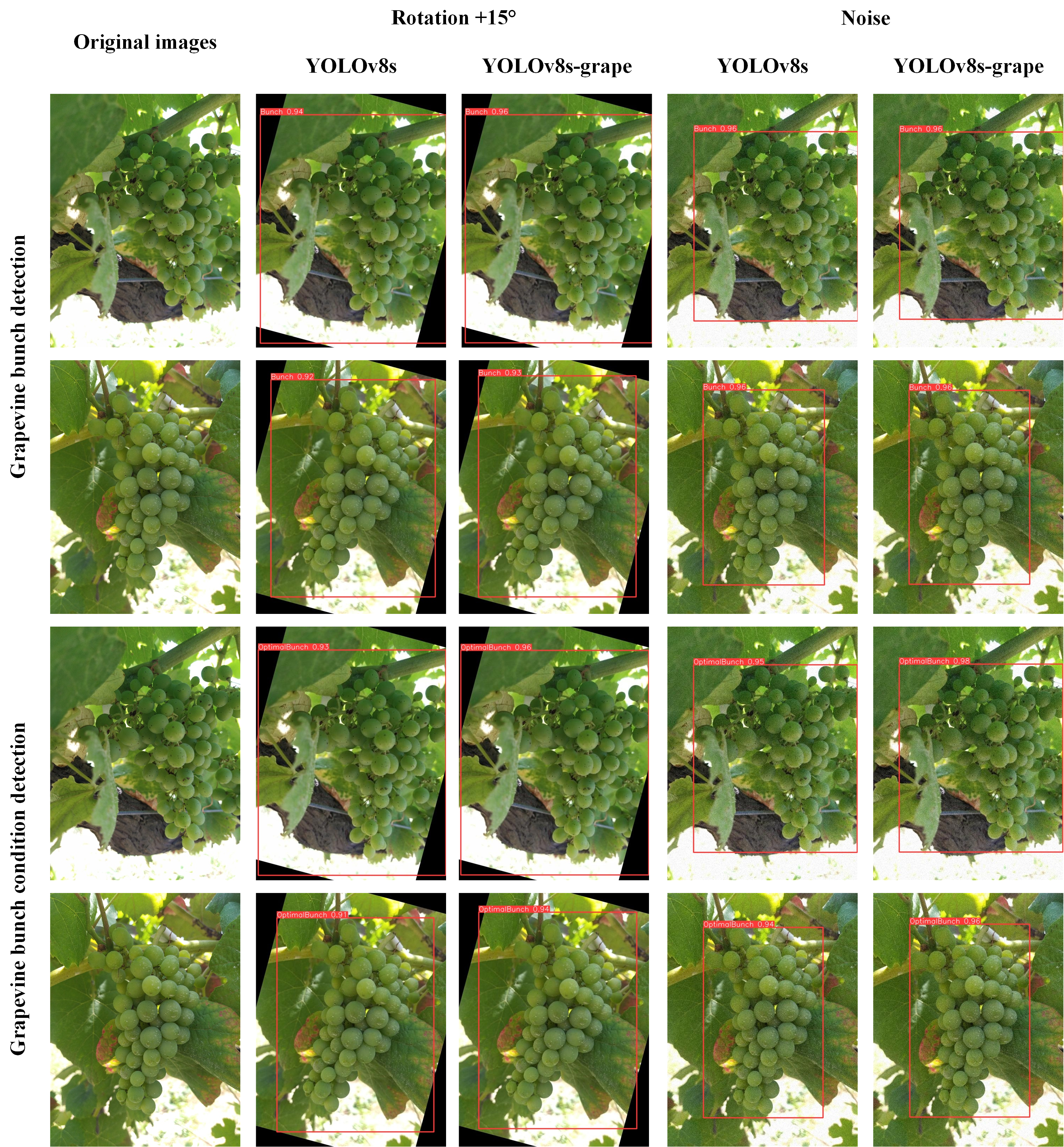

Supplement: Supplementary file 1 [file DataSheet_1.zip › New Data Sheet 1/Images/Supplementary Figure S4. The test results of YOLOv8s and the proposed method.tif]

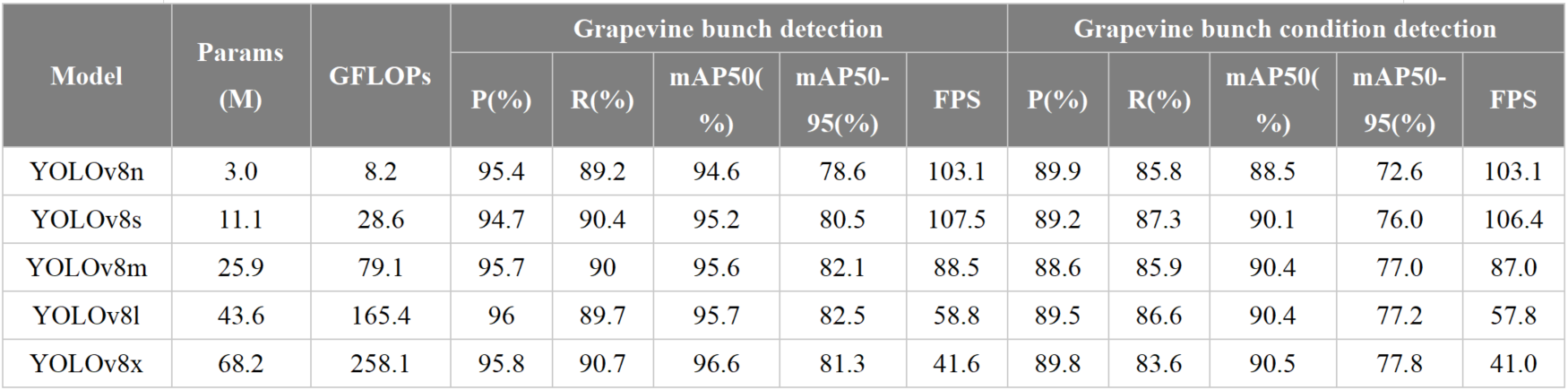

Supplement: Supplementary file 1 [file DataSheet_1.zip › New Data Sheet 1/Tables/Supplementary Table S1. Comparison of the baseline model selection.tif]

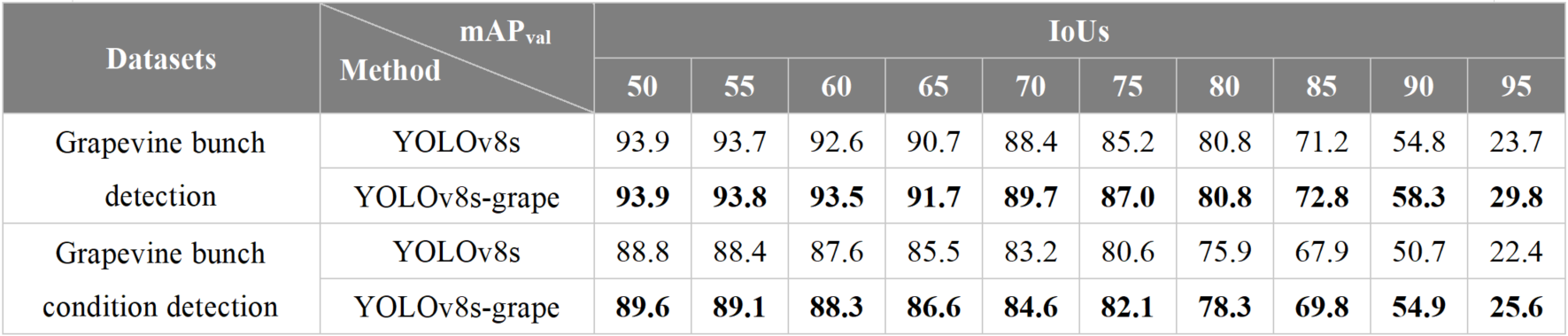

Supplement: Supplementary file 1 [file DataSheet_1.zip › New Data Sheet 1/Tables/Supplementary Table S2. Comparison of different IoUs of YOLOv8s and the proposed method.tif]
